# Combined lactic acidosis and ketoacidosis in a female diabetic patient with severe heart failure

**DOI:** 10.1097/XCE.0000000000000287

**Published:** 2023-07-04

**Authors:** Desire Nzomessi, Emmanuelle Massie, Karim Gariani, Raphael Giraud, Philippe Meyer

**Affiliations:** a Division of Cardiology; b Division of Endocrinology, Diabetology, Nutrition and Therapeutic Education; c Division of Intensive Care, University Hospital of Geneva, Geneva, Switzerland

**Keywords:** case report, ketoacidosis, lactic acidosis, metformin, SGLT2-inhibitors

## Abstract

SGLT2i are now recommended in a wide spectrum of indications including type 2 diabetes (T2DM), heart failure, and chronic kidney disease. This medication class is now available in combination with metformin, which is still a fundamental treatment in patients with T2DM. Despite excellent proven safety profile for both drugs, the expanding use of these agents in clinical practice may lead to an increased incidence of rare side effects, like metformin-associated lactic acidosis (MALA) and euglycemic diabetic ketoacidosis (EDKA), which can be life-threatening. A 58-year-old woman with T2DM and severe heart failure treated by metformin and empagliflozin developed progressive EDKA triggered by fasting that was also complicated by severe acute renal failure and MALA. She was successfully treated with intermittent hemodialysis. This case report highlights the importance of the recognition of rare, but very serious adverse effects due to combined metformin and SGLT2i therapy.

## Introduction

According to current guidelines, metformin and sodium-glucose cotransporter-2 inhibitors (SGLT2i) are considered first-line oral antidiabetic therapies in patients with type 2 diabetes (T2DM) and confirmed or at high risk of cardiovascular disease [1]. Moreover, clinical trials have demonstrated that SGLT2i decrease cardiovascular mortality and heart failure hospitalizations in patients with heart failure, independently of the presence of T2DM or left ventricular ejection fraction (LVEF) value [2–5]. SGLT2i are part of the four foundational therapies of heart failure with reduced (≤40%) ejection fraction [6], and they are also the first pharmacological agents improving clinical outcomes in patients with heart failure and LVEF > 40% [4,5]. Several combinations of metformin and SGLT2i are now available, enhancing the prescription of both of these agents. Despite more than 60 years of clinical experience with metformin and an excellent safety profile of SGLT2i in large randomized clinical trials, they can rarely lead to life-threatening complications due to high anion gap metabolic acidosis [7]. We report here a case of a female patient with advanced heart failure and T2DM that presented with severe combined metformin-associated lactic acidosis (MALA) and euglycemic diabetic ketoacidosis (EDKA) that was successfully managed with intermittent hemodialysis. Written informed consent was obtained from the patient.

## Case report

A 58-year-old female patient known for T2DM was admitted to our institution for breathlessness and confusion. Four months before, she had been diagnosed with an ST-elevation myocardial infarction due to an occluded circumflex artery treated by angioplasty and stenting. Chronic occlusion of the left anterior descending artery was not revascularized due to the absence of myocardial ischemia or viability on functional imaging. During follow-up, because of advanced heart failure with New York Heart Association functional class III and LVEF 25% despite maximum tolerated heart failure medical therapy, she underwent implantable cardioverter-defibrillator implantation and was listed for heart transplant. At that moment, T2DM was well controlled [glycated hemoglobin (HbA1c] 6.9%) and an impaired kidney function, which was stable compared with previously [estimated glomerular filtration rate (eGFR) 48 ml/min/1.73 m^2^]. Her medication included the following: aspirin 100 mg od, clopidogrel 75 mg od, atorvastatin 40 mg od, ezetimibe 10 mg od, metoprolol 100 mg od, sacubitril/valsartan 50 mg bid, spironolactone 25 mg od, empagliflozin 10 mg od, metformin 500 mg bid, and long-acting insulin 6 units od.

A few days before the admission to our hospital, the patient wanted to celebrate her birthday with her husband and drove 250 km by car to one the most touristic places in Switzerland, Interlaken. Unfortunately, all restaurants and hotels were closed because of the coronavirus disease 2019 pandemic and the couple finally had to return to Geneva late the same day without having been able to eat or drink. At night, the patient started to feel progressive symptoms of nausea, vomiting, diarrhea, anorexia, and abdominal pain. She could hardly eat or drink but continued her medications until hospital admission 4 days later.

Upon admission, the patient was confused, with Glasgow Coma Scale 11/15, and in respiratory distress. Vital signs were the following: blood pressure 134/85 mmHg; heart rate 90 bpm; respiratory rate 40/min; oxygen saturation 97% on room air; temperature 36.0 °C. There was no sign of congestion. Blood chemistry showed the following: sodium 135 mmol/l, potassium 6.4 mmol/l, creatinine 801 μmol/l, glucose 9 mmol/l. Arterial blood gases are shown in Table 1. Ketone bodies were elevated at 5.6 mmol/l and lactate at 17 mmol/l. Metformin level was 40.0 mg/l (normal values 0.1–1.3 mg/l). All oral antidiabetic medications were discontinued. The patient was admitted to the ICU and treated by IV bicarbonates and intermittent dialysis with rapid correction of acidosis and return to previous renal function level after 9 days. Oral antidiabetics were switched to glucagon-like peptide-1 receptor agonists and gliclazide, and the patient was discharged home after 4 weeks. She was successfully transplanted 5 months later.

**Table 1:** Blood gases during hospitalization

Blood gases	Normal values	Admission	After one session of intermittent dialysis	Before discharge
pH	7.35–7.45	6.72	7.37	7.47
PCO_2_ (kPa)	4.27–6.00	0.98	2.76	3.71
PO_2_ (kPa)	11.07–14.40	30	15.8	13.2
HCO3 (mmol/l)	22.0–26.0	3.4	12.9	20
Anion gap (mmol/l)	8–16	22.6	11.1	7
Lactate (mmol/l)	0.5–1.6	17	6.5	1.1
Beta-hydroxybutyrate (mmol/l)	<0.6	5.6	–	–
Glycemia (mmol/l)	4.1–6.0	9	8	4.5

## Discussion

Metformin has been the first-line therapy for patients with T2DM for decades, mainly because of its high efficacy in glycemic control (via multiple molecular targets) with a concomitant low risk of hypoglycemia [1]. Benign gastrointestinal symptoms such as diarrhea and nausea are the most frequent side effects, but MALA is a potentially fatal condition that has rarely been reported. Patients with liver/renal impairment, heart failure, and older age are more at risk of developing this complication while on treatment [7].

SGLT2i represent a new class of antidiabetic therapy improving glycemic control by enhancing urinary excretion of glucose [1]. The most serious side effect reported is EDKA, defined by relative euglycemia (<250 mg/dl/<11. mmol/l) with increased anionic gap metabolic acidosis (pH < 7.3, HCO3^−^ <18 mmol/l) and ketosis [8]. Glucosuria secondary to SGLT2i leads to decreased plasma glucose levels, which will in turn reduce insulin and increase glucagon secretion. The subsequent increased glucagon/insulin ratio stimulates lipolysis and ketone body production, and can therefore potentially lead to EDKA. The main triggers of EDKA are fasting, acute infection, discontinuation of insulin therapy (endogenous or exogenous), alcohol abuse, and severe dehydration. In the most recent randomized clinical trials in heart failure patients, the incidence of ketoacidosis ranged from 0% to 0.1% for empagliflozin and dapagliflozin [2–5].

Symptoms of lactic acidosis and ketoacidosis are similar and include nausea, vomiting, malaise, abdominal pain, and, in more severe cases, altered consciousness, Kussmaul breathing, and clinical signs of shock [9]. Our main hypothesis regarding our patient is that fasting precipitated ketone acid production, leading to anorexia, nausea, and vomiting, leading to hypovolemia, and secondary acute renal failure, which could be the cause of metformin accumulation that further enhanced lactic and ketone acidosis.

Very few cases in the literature describe the combination of MALA and EDKA in patients treated with metformin and SGLT2i. We found one case described in a patient taking canagliflozin and metformin who had gastroenteritis, and another in a patient treated by empagliflozin and metformin who suffered from acute renal failure due to high doses of celecoxib, and both were successfully treated with continuous renal replacement therapy (CRRT) and parenteral insulin/glucose infusion [9,10].

Initial management for MALA is based on administration of intravenous fluid and supportive care. There is no specific treatment, and sodium bicarbonate alone is generally not sufficient to correct acidosis [11]; however, in patients with hemodynamic instability and organ failure, early CRRT appears to be safe and effective [12]. Regarding EDKA, crystalloid fluid resuscitation (e.g. PlasmaLyte, Ringer’s Lactate) with 1–2 l infusion during the first 1–2 h is the first step. Insulin at a rate of 0.05–0.1 units/kg/h should be administrated concurrently with Dextrose 5% for management of ketosis and to avoid hypoglycemia. Moreover, low serum potassium should be corrected to reach a level above 3.5 mEq/l before insulin infusion is initiated, and electrolytes/glucose should be monitored every hour [8].

From a prevention perspective, patients with severe renal failure remain at high risk of MALA during treatment with metformin, therefore its prescription should be avoided when eGFR is below 45 ml/min/1.73 m^2^ [13]. Regarding EDKA, because SGLT2i have a longer half-life (>12 h) with a long bioavailability [7], the European Medicines Agency proposes stopping SGLT2i 3 days before surgery under general anesthesia, and it should also be temporarily discontinued in patients with potential triggers for ketoacidosis including fever, vomiting, or diarrhea [14]. Sick day rules cards have been developed to inform patients with chronic diseases which medicines (including among others metformin and SGTL2i) to temporarily stop during a dehydrating illness [15]. Once EDKA has been diagnosed, it is recommended to discontinue SGLT2i forever [14]. There is no scientific consensus on the resumption of metformin after MALA, but in our case, we decided to stop it definitely as well.

### Conclusion

This case illustrates the potentially serious risks associated with metformin and SGLT2i. Because of their association in one unique pill in addition to the growing evidence around the benefits of SGLTi, these drugs are likely to be more and more prescribed, and thus, clinicians need to be aware of MALA and EDKA in order to counsel their patients better, diagnose these conditions early when they occur, by measuring ketones and lactates in the presence of metabolic acidosis, and provide adequate and rapid management in order to minimize complications. Finally, we suggest caution when combining these drugs in patients with potential risk factors or triggers for MALA/EDKA.

## Acknowledgements

### Conflicts of interest

There are no conflicts of interest.
